# Supplementary material for: Improvement in Migration Resistance of Hydroxyl-Terminated Polybutadiene (HTPB) Liners by Using Graphene Barriers
Source: Polymers (Basel). 2022 Nov 30;14(23):5213. doi: 10.3390/polym14235213 (PMC9740540; doi:10.3390/polym14235213)
Supplement: Supplementary file 1 [file polymers-14-05213-s001.zip › polymers-2023184-supplementary.pdf]

Preparation of GO

First, add 6 g graphite powder and 3 g $NaNO_3$ into the beaker, and add 150 ml H_2SO_4 under the ice bath. Then 18 g $KMnO_4$ was added in portions. The temperature was raised to 35 °C in a water bath. After 2h, 200 mL of deionized water was added, and stirring was continued for 2h. The temperature was then raised to 98 °C and 400 mL of deionized water was added to dilute the solution. Add a small amount of 30%H_2O_2 dropwise until the solution turns bright yellow. Wash away metal ions with dilute hydrochloric acid. Centrifuge and wash until the solution is neutral. Freeze it for later use.

Preparation of TGO

Weigh 0.2 g of the prepared GO, use anhydrous DMF solution to prepare a 1 mg/mL suspension, ultrasonically disperse the suspension for 3 hours, and stir at 80 °C after the ultrasonication is completed. Slowly add 1 g of toluene TDI into the reaction solution dropwise. One drop of DBTL was reacted with N_2 protection for 5 h. After the reaction, the product was collected by filtration, washed with DMF to remove unreacted TDI, and dried to obtain TGO.

Preparation of the bonded samples

First, the titanium alloy sheets (100 × 25 × 1.5 mm) were physically sandblasted, and then cleaned with an acetone/ethanol (1/1, v/v) solution to remove contaminants on the metal surface. The single-lap test dimensions are shown in Figure S1. The adhesive used in this test was an incompletely cured liner slurry. Take a certain amount of liner slurry and coat it on the interface of the titanium plate. After bonding the sample, fix it with a dovetail clip, and put it into a vacuum drying oven together with the lining sample to solidify. The rubber compound overflowing around, the curing process of the sample is shown in Figure S2.

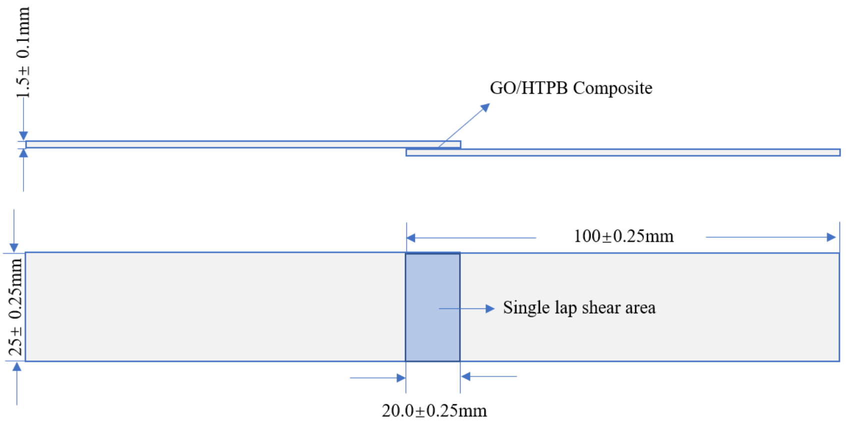

Figure S1 Schematic diagram of a single-lap sample

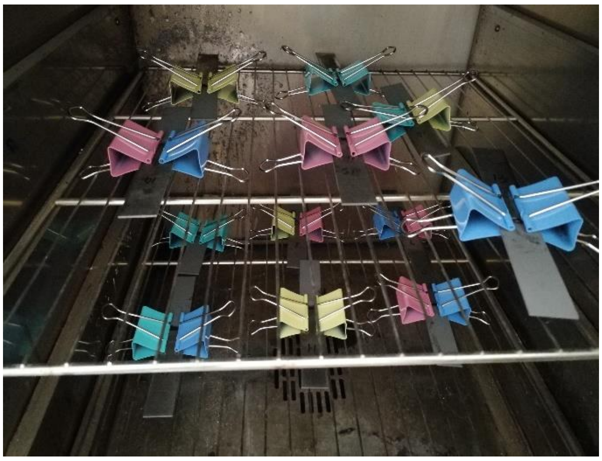

Figure S2 Schematic diagram of single-lap sample curing

Performance comparison of various liners

Table S1. Summary of performance comparison of various liners

Properties / Type	Migration concentration	Migration dynamics		Migration thermodynamics		Mechanical properties	
		Migration coefficient	R^2	Migration activation energy	r^2	tensile strength	bond strength
Pure HTPB	86.27	3.93E-7	0.994	27.69	0.981	0.18	0.25
GH	66.19	3.37E-8	0.989	42.82	0.951	0.27	0.95
TGH	58.45	2.35E-8	0.989	45.01	0.992	0.34	1.56
GAH	62.84	6.29E-8	0.990	38.71	0.982	1.12	1.72
